# On the trapdoor spiders of Mexico: description of the first new species of the spider genus *Aptostichus* from Mexico and the description of the female of *Eucteniza zapatista* (Araneae, Mygalomorphae, Euctenizidae)

**DOI:** 10.3897/zookeys.641.10521

**Published:** 2016-12-16

**Authors:** Alejandro Valdez-Mondragón, Mayra R. Cortez-Roldán

**Affiliations:** 1 Conacyt Research Fellow, Laboratory of Arachnology. Laboratorio Regional de Biodiversidad y Cultivo de Tejidos Vegetales del Instituto de Biología UNAM, sede Tlaxcala. Contiguo FES-Zaragoza Campus III, Ex Fábrica San Manuel de Morcom s/n, San Miguel Contla, C.P. 90640, Municipio de Santa Cruz Tlaxcala, Tlaxcala, Mexico; 2 Colección Nacional de Arácnidos (CNAN), Departamento de Zoología, Instituto de Biología, Universidad Nacional Autónoma de México. 3er. Circuito exterior s/n. Apartado Postal 70-153, C.P. 04510, Ciudad Universitaria, Coyoacán, Ciudad de México, Mexico

**Keywords:** Aptostichus, Eucteniza, Euctenizidae, new species, Mexico, taxonomy

## Abstract

A new species of the spider genus *Aptostichus* Simon, 1891 is described from a cave in Huautla de Jiménez, Oaxaca, Mexico: *Aptostichus sabinae* **sp. n.** This species represents the first new species described from Mexico and the southernmost record in North America for the genus so far. *Aptostichus sabinae* **sp. n.** represents the forty-first species described for the genus, which has the highest species diversity in the family Euctenizidae. *Eucteniza zapatista* is redescribed based on five new males and the first known female from the Parque Nacional La Malinche (PNLM), Tlaxcala Mexico. *Eucteniza zapatista* is the fourth species of the genus where a female is known, and one of fourteen species described for the genus to date.

## Introduction

Currently, the trapdoor spider family Euctenizidae Raven, 1985 comprises 75 species described in seven genera: *Apomastus* Bond & Opell, 2002; *Aptostichus* Simon, 1891; *Entychides* Simon, 1888; *Eucteniza* Ausserer, 1875; *Myrmekiaphila* Atkinson, 1886; *Neoapachella* Bond & Opell, 2002; and *Promyrmekiaphila* Schenkel, 1950 (Bond and Godwin 2013, World Spider Catalog 2016). Previously, Euctenizinae was a subfamily that included all the North American cyrtaucheniid genera (Raven 1985); however, morphological and molecular cladistic analyses (Bond and Opell 2002, Bond and Hedin 2006, Bond et al. 2012, Hedin and Bond 2006), demonstrated that the family Cyrtaucheniidae Simon, 1889 was polyphyletic and Euctenizinae was recovered as monophyletic group, and was elevated posteriorly to family status by Bond et al. (2012). Among the euctenizid genera, *Aptostichus* has the highest species diversity with 40 species not including the species described herein (Bond 2012, World Spider Catalog 2016). The last taxonomic revision and phylogeny of the genus was made by Bond (2012), where 33 new species were described and classified in four species groups: Atomarius, Hesperus, Simus, and sierra. All the species of *Aptostichus* are distributed in the southwestern United States, with only three records in Mexico, in Baja California (World Spider Catalog 2016). The genus *Eucteniza*, which previously was the type genus for the cyrtaucheniid subfamily Euctenizinae (Raven 1985, Bond and Hedin 2006), currently comprises 14 species. The taxonomic revision of the genus was made by Bond and Godwin (2013), where 12 new species were described. Most of the species are distributed in Mexico (13 species), mainly from Baja California, along to the Sierra Madre Oriental and central part of the Transmexican Volcanic Belt (Bond and Godwin 2013: fig. 1), and two species from Texas, United States: *Eucteniza ronnewtoni* Bond & Godwin, 2013, and *Eucteniza relata* (O. Pickard-Cambridge, 1895) which is distributed widely throughout Texas and Northern Mexico (Bond and Godwin 2013, World Spider Catalog 2016). Like most of the trapdoor spiders, the specimens are difficult to collect, and *Aptostichus* and *Eucteniza* are no exception. Specimens are rare, even in biological collections. Most of the species are described based only on male specimens. For example, in *Eucteniza*, only three species of 14 are described based on both sexes (World Spider Catalog 2016). The type species for the genus was originally described based on a juvenile specimen, *Eucteniza atoyacensis* Bond & Opell, 2002, considered by Bond and Godwin (2013) as a nomen dubium ((*Eucteniza mexicana* (O. Pickard-Cambridge, 1895)). In this work, a new species of the genus *Aptostichus* is described based on a male collected from a cave in Oaxaca, Mexico; additionally the female of *Eucteniza zapatista* is described for the first time from Parque Nacional La Malinche (PNLM), Tlaxcala, Mexico.

## Material and methods

The specimens were collected and deposited in 80% ethanol and, labeled with their complete field data. For the descriptions the specimens were observed using a Zeiss Discovery.V8 stereoscope. A Zeiss Axiocam 506 color camera attached to a Zeiss AXIO Zoom.V16 stereoscope was used to photograph the different structures of specimens. All structures photographed under the stereoscope were submerged in gel alcohol (available commercially as a hand cleaner). The firm consistency of the gel allows for the immobilization and positioning of the structure to be photographed. The structure suspended in the gel alcohol was covered with 80% liquid ethanol to minimize diffraction during examination and photography. All measurements in the descriptions are in millimeters (mm). Photographs were edited with Adobe Photoshop CS6.

The holotype specimen of *Aptostichus sabinae* sp. n. is deposited with its collection code in the Colección Nacional de Arácnidos (**CNAN**) of the Instituto de Biología UNAM (**IBUNAM**), Mexico City. The holotype of *Eucteniza zapatista* Bond & Godwin, 2013 was previously deposited in the American Museum of Natural History (**AMNH**), New York, U.S.A. The specimens of *Eucteniza zapatista* used for this work, are deposited with their collections codes in the collection of the Laboratory of Arachnology (**LATLAX**), Laboratorio Regional de Biodiversidad y Cultivo de Tejidos Vegetales of the Instituto de Biología UNAM (**IBUNAM**), Tlaxcala City. Morphological nomenclature and measurements follow Bond (2012) and Bond and Godwin (2013).

Abbreviations used in the description are:

**B** bulb;

**Cl, Cw** carapace length and width (widest part);

**Cy** cymbium;

**E** embolus;

**LBl, LBw** labium length and width taken from the longest and widest points, respectively;

**PTl, PTw** male palpal tibia length and width (widest part in dorsal view);

**STRl, STRw** sternum length and width (widest part);

**v** ventral;

**p** prolateral.

## Taxonomy

### Family Euctenizidae Raven, 1985

#### Genus Aptostichus

Taxon classificationAnimaliaAraneaeEuctenizidae

Simon, 1891

##### Type species.

*Aptostichus atomarius* Simon, 1891.

##### Diagnosis.

For updated diagnosis of the genus see Bond (2012): 29.

##### General description.

For updated description of the genus see Bond (2012): 29.

##### Species groups.

Atomarius, Hesperus, Simus, and Sierra (Bond 2012).

##### Composition.

*Aptostichus aguacaliente* Bond, 2012; *Aptostichus angelinajolieae* Bond, 2008; *Aptostichus anzaborrego* Bond, 2012; *Aptostichus asmodaeus* Bond, 2012; *Aptostichus atomarius* Simon, 1891; *Aptostichus barackobamai* Bond, 2012; *Aptostichus bonoi* Bond, 2012; *Aptostichus cabrillo* Bond, 2012; *Aptostichus cahuilla* Bond, 2012; *Aptostichus cajalco* Bond, 2012; *Aptostichus chavezi* Bond, 2012; *Aptostichus chemehuevi* Bond, 2012; *Aptostichus chiricahua* Bond, 2012; *Aptostichus dantrippi* Bond, 2012; *Aptostichus derhamgiulianii* Bond, 2012; *Aptostichus dorothealangeae* Bond, 2012; *Aptostichus edwardabbeyi* Bond, 2012; *Aptostichus elisabethae* Bond, 2012; *Aptostichus fisheri* Bond, 2012; *Aptostichus fornax* Bond, 2012; *Aptostichus hedinorum* Bond, 2012; *Aptostichus hesperus* (Chamberlin, 1919); *Aptostichus huntington* Bond, 2012; *Aptostichus icenoglei* Bond, 2012; *Aptostichus isabella* Bond, 2012; *Aptostichus killerdana* Bond, 2012; *Aptostichus lucerne* Bond, 2012; *Aptostichus mikeradtkei* Bond, 2012; *Aptostichus miwok* Bond, 2008; *Aptostichus muiri* Bond, 2012; *Aptostichus nateevansi* Bond, 2012; *Aptostichus pennjillettei* Bond, 2012; *Aptostichus sabinae* sp. n., *Aptostichus sarlacc* Bond, 2012; *Aptostichus satleri* Bond, 2012; *Aptostichus serrano* Bond, 2012; *Aptostichus sierra* Bond, 2012; *Aptostichus simus* Chamberlin, 1917; *Aptostichus sinnombre* Bond, 2012; *Aptostichus stanfordianus* Smith, 1908; and *Aptostichus stephencolberti* Bond, 2008. Total: 41 species.

##### Distribution.

United States, Mexico.

#### Aptostichus sabinae sp. n.

Taxon classificationAnimaliaAraneaeEuctenizidae

http://zoobank.org/E78270D0-B3A8-4BC7-B2E3-FF617CBFA6A3

Figs 1–4 , 5–7 , 8–12 , 13–20

##### Type material.

MEXICO: Oaxaca: 1♂ holotype (CNAN-T1121) from Cueva Li Nita (lat 18.14767°, lon -96.79844°, 1919 m), Municipio Huautla de Jiménez, 12-April-2014, J. Mendoza, J. Cruz, S. Davlantes, M. Minkton Cols.

##### Etymology.

This species is dedicated to the María Sabina Magdalena García "María Sabina", a famous Mazatec shaman due to her traditional knowledge of healing and ceremonial use of hallucinogenic mushrooms who was born in 1894 in Huautla de Jiménez (municipality of the type locality), Oaxaca, Mexico.

##### Diagnosis.

Males are easily distinguished from the other known species of *Aptostichus* by the combination of the following characters: 1) a very long, slender and sigmoidal unique embolus (Figs 8, 9, 10, 12); 2) massive ventral and prolateral spines on the palpal tibiae (Figs 8, 11); 3) retrolateral-ventral small finger-shaped projection on metatarsus I (Fig. 19, arrows Figs 17, 18); 4) by having many spines on tibiae and metatarsi III and IV (Fig. 20); and 5) a unique dorsal opisthosomal pattern (Fig. 1).

**Figures 1–4.**
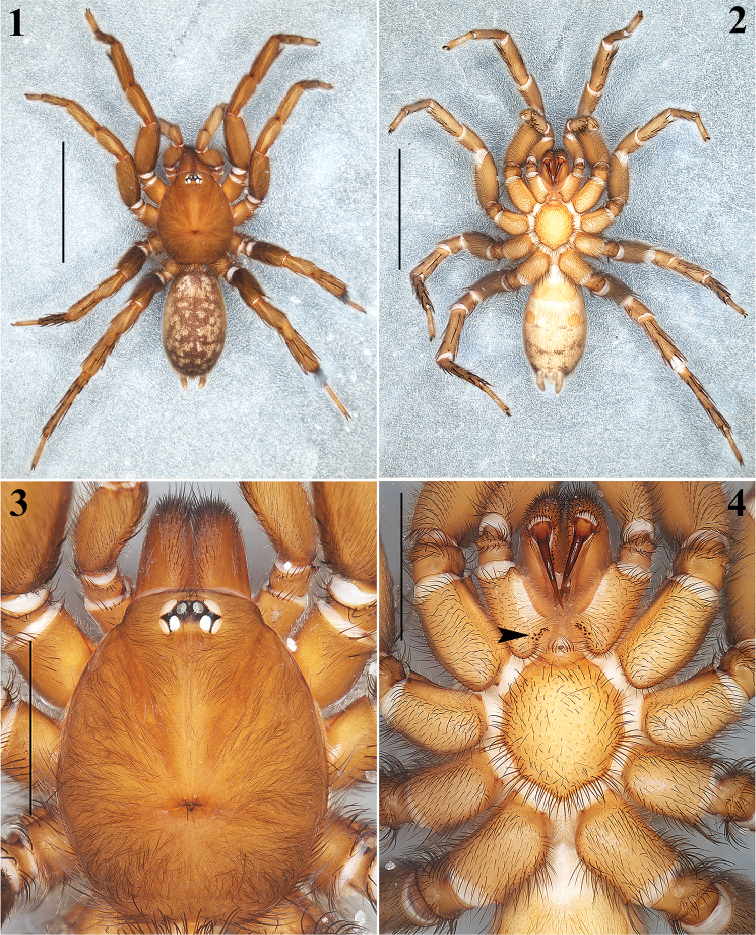
*Aptostichus sabinae* sp. n. Male holotype: **1–2** Habitus, dorsal and ventral views respectively **3** Carapace, dorsal view **4** Prosoma, ventral view showing coxae, sternum, labium and endites (arrow indicates cuspules). Scale bars 0.5 mm (**1**, **2**), 0.2 mm (**3**, **4**).

**Figures 5–7.**
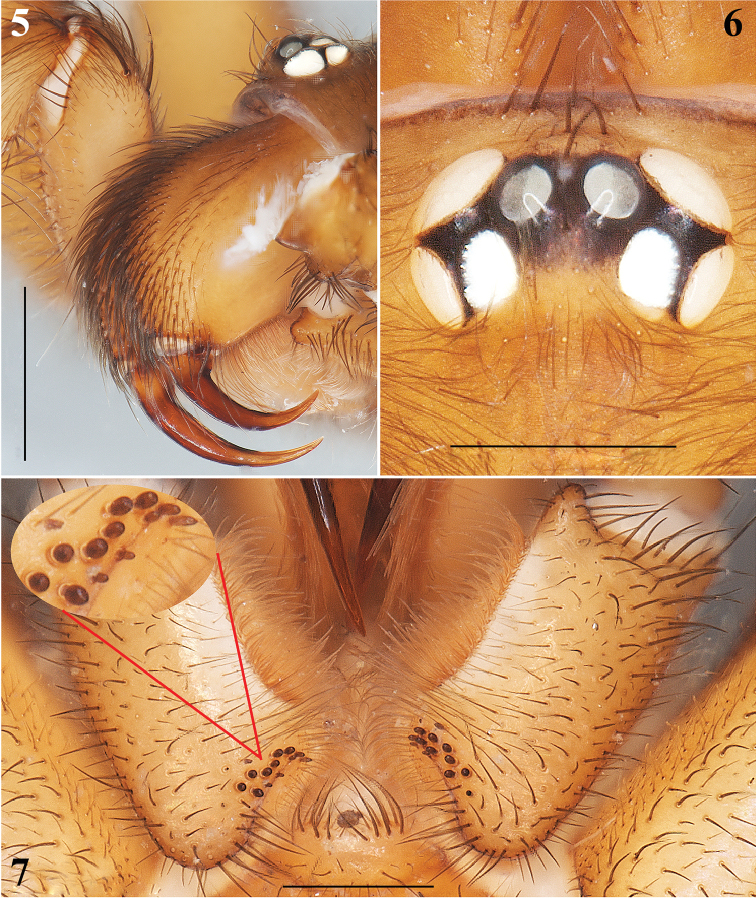
*Aptostichus sabinae* sp. n. Male holotype: **5** Chelicerae, lateral view **6** Ocular region **7** Endites, ventral view; detail of the cuspules. Scale bars 0.5 mm (**6**, **7**), 1 mm (**5**).

**Figures 8–12.**
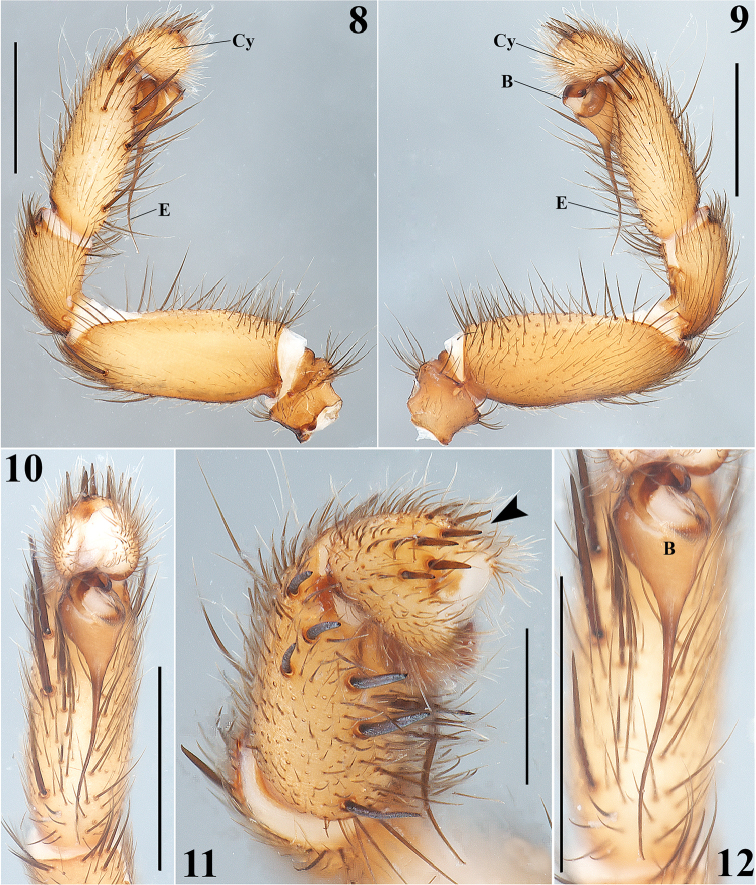
*Aptostichus sabinae* sp. n. Male holotype: **8–9** Left palp, prolateral and retrolateral views respectively **10** Left palp, ventral view **11** Left palp, prolateral-apical view (arrow indicates apical spines on cymbium) **12** Detail of the bulb and embolus, ventral view. Scale bars 0.5 mm (**11**), 1 mm (**8–10**, **12**).

**Figures 13–20.**
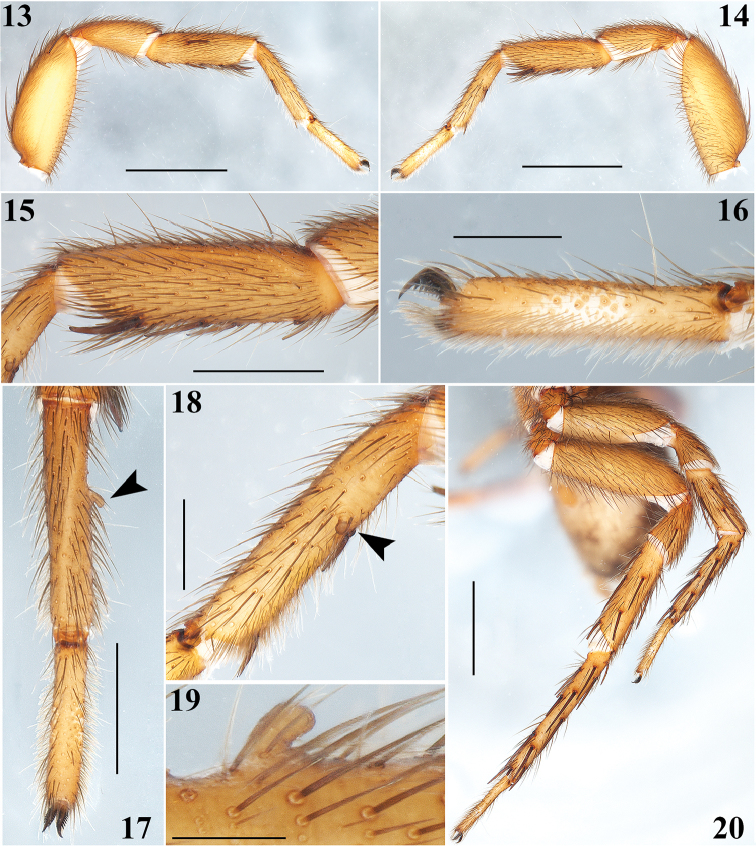
*Aptostichus sabinae* sp. n. Male holotype: **13–14** Left leg I, prolateral and retrolateral views respectively **15** Tibia I, retrolateral view **16** Tarsus I, retrolateral view**17** Metatarsus and tarsus I, dorsal view (arrow indicates the small finger-shaped projection on retrolateral-ventral part of metatarsus) **18** Metatarsus I, retrolateral view (idem) **19** Detail of the small finger-shaped projection on retrolateral-ventral part of metatarsus I. **20**, Spination pattern on legs III and IV. Scale bars 0.2 mm (**19**), 0.5 mm (**16**, **18**), 1 mm (**15**, **17**), 2 mm (**13**, **14**, **20**).

##### Description.

**Male (holotype)**: Specimen collected manually, preserved and observed in 80% ethanol. *Measurements*: Total length (prosoma + opisthosoma) 8.30. Carapace 3.84 long, 3.12 wide. Clypeus length 0.18. Diameter of AME 0.13, ALE 0.25, PME 0.18, PLE 0.23. Labium: LBl 0.23, LBw 0.53. Sternum: STRl 1.85, STRw 1.65. Leg lengths: I femur 2.75/ patella 1.80/ tibia 2.2/ metatarsus 1.88/ tarsus 1.36/ total 9.99; II- 2.50/ 1.64/ 1.88/ 1.68/ 1.24/ 8.94; III- 2.25/ 1.32/ 1.76/ 2.40/ 1.28/ 9.01; IV- 3.00/ 1.60/ 2.48/ 3.50/ 1.52/ 12.10. Leg formula: 4-1-3-2. *Prosoma*: Carapace longer than wide, with surface smooth, setose, pyriform shaped, light brown coloration (Figs 1, 3). Ocular region slightly elevated (Figs 3, 5, 6). Foveal groove slightly deep (Fig. 3). AER slightly procurved, PER slightly recurved (Figs 3, 6). Largest ALE, smallest AME (Fig. 6). Sternum longer than wide, nonagonal shaped, orange, more setose towards posterior margin, without sigilla (Fig. 4). Labium wider than long, orange, with long setae anteriorly, without cuspules (Fig. 7). Endites long and setose, with an apical-prolateral conspicuous conical apophysis, with a patch of 11-13 small cuspules on proximal inner part on each endite (Fig. 7). *Chelicerae*: Promargin furrow with 6 teeth, retromargin furrow with single row of very long setae. Rastellum consists of numerous long setae, but without stout spines. *Opisthosoma*: Longer than wide, setose, beige, with irregular brown pattern dorsally; ventrally with brown undefined lines, close to spinnerets (Figs 1, 2). Spinnerets beige. PMS small and rounded apically, single segment, with spigots. PLS long and conical apically, all 3 segments with spigots: basal segment length > median segment > distal segment. *Legs*: Light tarsal scopulae on all legs (Fig. 16). Tibiae, metatarsi and tarsi with trichobothria: Tibiae I-IV: two prolateral-dorsal rows with 9 trichobothria each, distal ones becoming larger; metatarsi I and II: one dorsal row with 12; metatarsi III: one dorsal row with 15; metatarsi IV: one dorsal row with 19; tarsi I: slightly staggered dorsal row with 10; tarsi II: slightly staggered dorsal row with 12; tarsi III: slightly staggered dorsal row with 10; tarsi IV: slightly staggered dorsal row with 11. Legs spination pattern: Tibiae I: v(2+2+2) (one of the last spines -retrolateral- is massive) (Figs 14, 15), p(1) (Fig. 13); tibiae II: v(2+2+2), p(1+1); metatarsi I: v(2+1); metatarsi II: v(1+2+2), p(1). Leg III and IV spination pattern is illustrated in Figure 20. *Pedipalps*: Articles pale orange, slender, femora and tibiae with long setae ventrally (Figs 8, 9). Patellae with distal dorsal-prolateralspine. Tibiae with massive ventral and prolateral spines (Figs 8, 10–12). Cymbium with seven dorsoapical spines (arrow, Fig. 11). Bulb pyriform, turned ventrally toward a concavity on ventral part of tibiae (Figs 8, 9, 10, 12). Embolus long, slender and sigmoidal, almost with the same length as tibiae (Figs 8, 9, 10, 12).

**Female.** Unknown.

##### Remarks.

*Aptostichus sabinae* sp. n. resembles *Aptostichus asmodaeus* (Bond 2012: figs 127–132), from Contra Costa County, Mount Diablo State Park, California mainly in the shape of the retrolateral-ventral small finger-shaped projection on metatarsi I (arrows Figs 17, 18; Bond 2012: fig. 128). However, the spination pattern in leg I, the embolus and bulb shape (Figs 10, 12), the spination pattern on the ventral and prolateral part of palps tibiae (Fig. 11) (absent in *Aptostichus asmodaeus*; Bond 2012: figs 131, 132), and the opisthosoma dorsal pattern differ in both species (Bond 2012: figs 127–132). Following Bond (2012) and the synapomorphies that support each species groups, *Aptostichus sabinae* sp. n. does not fit into any of the groups. The *sierra* species group composed by four species is supported by two synapomorphies: long sternum and a long male metatarsus I (Bond 2012: figs 337, 338, 340), in *Aptostichus sabinae* the sternum is nonagonal shaped (Fig. 4) and the male metatarsus I is shorter (Figs 13, 14). The *simus* species group composed by eight species and supported by six synapomorphies: 1) absence of cuspules on male endites, present in *Aptostichus sabinae* (Fig. 7); 2) male palpal tibia stout (Bond 2012: figs 278, 287), in *Aptostichus sabinae* the palpal tibia is thinner (Figs 8, 9); 3) male palpal tibia spines short and positioned retrolaterally (Bond 2012: figs 278, 287), in *Aptostichus sabinae* the palpal tibia spines are long, scattered and positioned prolaterally (Figs 8, 9); 4) stout embolus (Bond 2012: figs 277, 306), *Aptostichus sabinae* has a long and thin embolus (Figs 10, 12); 5) embolus is dorsal - ventrally compressed (Bond 2012: figs 277), in *Aptostichus sabinae* is not (Figs 8, 9, 10, 12); and 6) retrolateral, distal most aspect of the cymbium formed as a distinct process (Bond 2012: fig. 277), absent in *Aptostichus sabinae* (Fig. 10). The *hesperus* species group, composed by thirteen species, is supported mainly by an offset retrolaretal rastellar spine (Bond 2012: fig. 189), which is absent in *Aptostichus sabinae* (Fig. 5). Also, four characters support the monophyly of this species group: 1) lighter carapace and 2) abdominal coloration, whereas in *Aptostichus sabinae* both colorations are darker than the other species of the group (Figs 1, 3); and 4) long and 5) sinuous spermathecal stalk, is unknown in *Aptostichus sabinae*. Finally, the *atomarius* species group, the most diverse and composed by fifteen species, is supported by three weak synapomorphies: 1) heavy carapace pubescence (Bond 2012: figs 101, 113), 2) dense female tarsal scopulae (38) and a distinct secondary spermathecal bulb. However, the carapace of *Aptostichus sabinae* has a slight carapace pubescence (Fig. 3), and the spermathecal bulb is unknown so far. Because to the synapomorphies explained above and mostly of them absent in *Aptostichus sabinae*, its placement within any of the species group proposed by Bond (2012) is uncertain. For that reason, we consider this new species as *inserta sedis* until the female of the species and more data and mainly new species from Mexico can be collected.

##### Natural history.

The holotype specimen was hand collected inside a cave, in a temperate forest at 1919 m of elevation. Although the specimen was collected in a cave, it does not present any troglomorphism or adaptation to cave life, and so might be considered a trogloxene.

##### Distribution.

Known only from the type locality (Fig. 53).

#### Genus Eucteniza

Taxon classificationAnimaliaAraneaeEuctenizidae

Ausserer, 1875

##### Type species.

*Eucteniza mexicana* Ausserer, 1875.

##### Diagnosis.

For updated diagnosis of the genus see Bond and Godwin (2013): 36.

##### General description.

For updated description of the genus see Bond and Godwin (2013): 36.

##### Composition.

*Eucteniza cabowabo* Bond & Godwin, 2013; *Eucteniza caprica* Bond & Godwin, 2013; *Eucteniza chichimeca* Bond & Godwin, 2013; *Eucteniza coylei* Bond & Godwin, 2013; *Eucteniza diablo* Bond & Godwin, 2013; *Eucteniza golondrina* Bond & Godwin, 2013; *Eucteniza hidalgo* Bond & Godwin, 2013; *Eucteniza huasteca* Bond & Godwin, 2013; *Eucteniza mexicana* Ausserer, 1875; *Eucteniza panchovillai* Bond & Godwin, 2013; *Eucteniza relata* (O. Pickard-Cambridge, 1895); *Eucteniza ronnewtoni* Bond & Godwin, 2013, *Eucteniza rosalia* Bond & Godwin, 2013; and *Eucteniza zapatista* Bond & Godwin, 2013. Total: 14 species.

##### Distribution.

Mainly from Baja California, along to the Sierra Madre Oriental and central part of the Transmexican Volcanic Belt (Bond and Godwin 2013: fig. 1), and with only one described species from Texas, United States.

#### Eucteniza zapatista

Taxon classificationAnimaliaAraneaeEuctenizidae

Bond & Godwin, 2013

Figs 21–24 , 25–27 , 28–31 , 32–39 , 40–44 , 45–49 , 50–51

Eucteniza zapatista Bond & Godwin, 2013: 54, f. 48–52 (Dm)

##### Type data.

MEXICO: Puebla: 1♂ holotype (EU012) (not examined), from Paso de Cortés (lat 19.1167°, lon -98.76676°, 3000 m), 18-July-1943, C. Bolivar Col. Holotype deposited in AMNH.

##### Material examined.

MEXICO: Tlaxcala: 4♂♂ (LATLAX-Ara0031) (pitfall traps) from 1.5 km al Oeste de la Estación Científica del Parque Nacional La Malinche (PNLM) (lat 19.24544°, lon -98.00336°, 3250 m), Municipio Ixtenco, 25-April-2016, A. Valdez, M. Cortez, A. Juárez Cols. 1♂ (LATLAX-Ara0033) from Carretera Perimetral con entronque Albergue IMSS Parque Nacional La Malinche (PNLM), Municipio Ixtenco, 4-May-2016, A. Ramírez Col. 1♀ (LATLAX-Ara0032) (hand collected) from Parque Nacional La Malinche (PNLM) (hand collected), El Pasaje (lat 19.25304°, lon - 97.97942°, 3030 m), Municipio Ixtenco, 03-July-2016, V. Jiménez, A. Díaz Cols.

##### Diagnosis.

Bond and Godwin 2013: “Male *Eucteniza zapatista* Bond and Godwin, 2013 leg I morphology is similar to *Eucteniza diablo* Bond and Godwin, 2013; however it lacks tarsal spines and has a more inflated or swollen tibia (Figs 32, 33, 36, 40–44). Males can be further distinguished from all other species by having an extensive patch of spines on the retrolateral distal aspect of the palpal tibia (Figs 28–30)”. Also, ventrally tibia I with very stout and paired megaspines, close each other (Figs 36, 38). Females with similar spermathecae to *Eucteniza diablo*, however in *Eucteniza zapatista* the spermathecae has a dark stalk and the bulbs with porous sculpture (Fig. 49), whereas in *Eucteniza diablo* only the basal part of the bulbs is dark and the bulbs lack porous sculpture (Bond and Godwin 2013: fig. 36).

**Figures 21–24.**
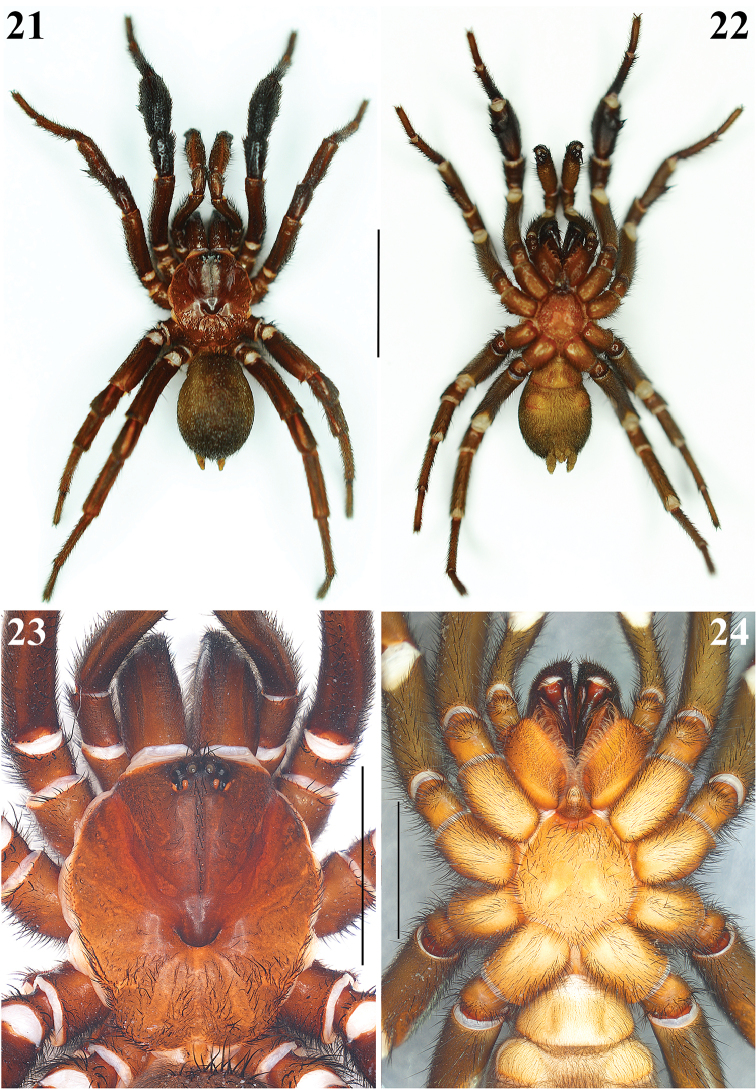
*Eucteniza zapatista* Bond & Godwin, 2013. Male: **21–22** Habitus, dorsal and ventral views respectively **23** Carapace, dorsal view **24** Prosoma, ventral view showing coxae, sternum, labium, and endites. Scale bars 0.5 mm (**23**, **24**), 1 mm (**21**, **22**).

**Figures 25–27.**
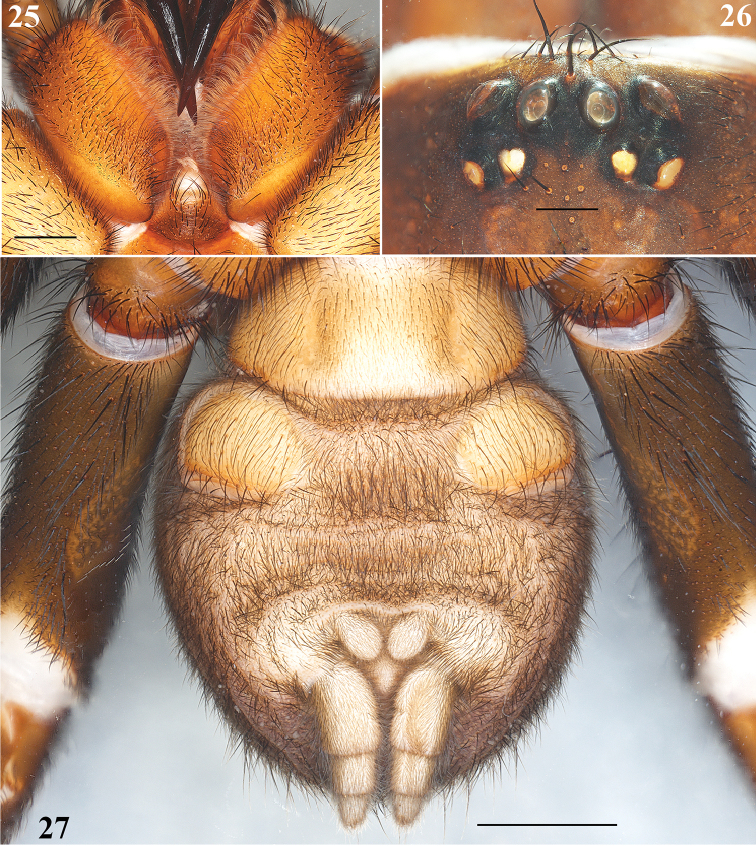
*Eucteniza zapatista* Bond & Godwin, 2013. Male: **25** Labium and endites, ventral view **26** Ocular region **27** Opisthosoma, ventral view, showing spinnerets. Scale bars 0.5 mm (**26**), 1 mm (**25**), 2 mm (**27**).

**Figures 28–31.**
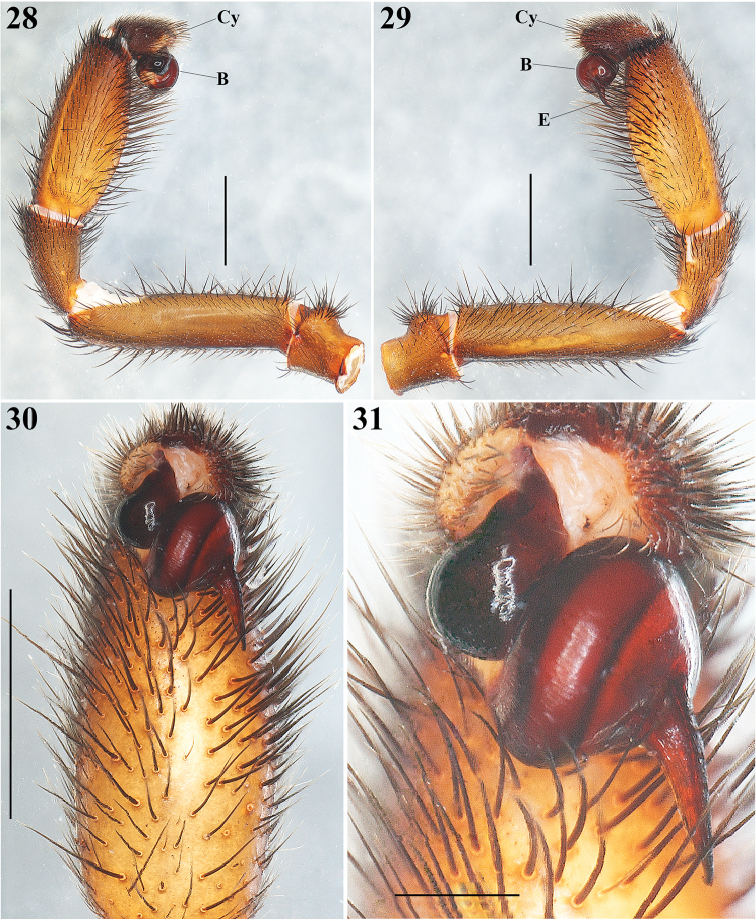
*Eucteniza zapatista* Bond & Godwin, 2013. Male: **28–29** Left palp, prolateral and retrolateral views respectively **30** Left palp, ventral view **31** Detail of the bulb and embolus, ventral view. Scale bars 0.5 mm (**31**), 2 mm (**28–30**).

**Figures 32–39.**
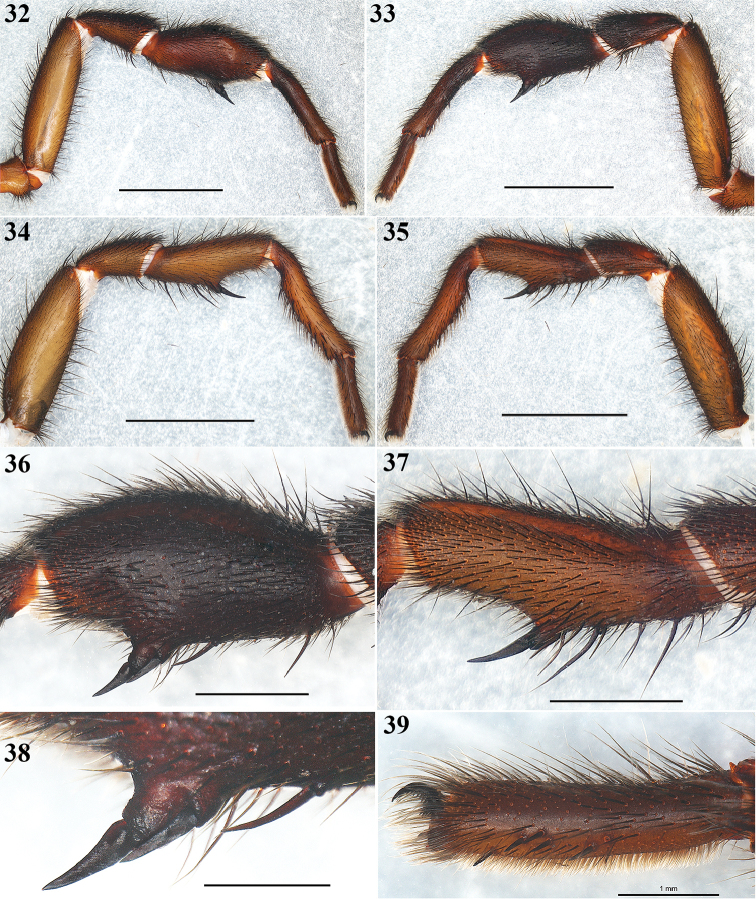
*Eucteniza zapatista* Bond & Godwin, 2013. Male: **32–33** Left leg I, prolateral and retrolateral views respectively **34–35** Left leg II, prolateral and retrolateral views respectively **36** Tibia I, retrolateral view **37** Tibia II, retrolateral view **38** Detail of the paired megaspines on tibia I **39** Scopulae on tarsus I, retrolateral view. Scale bars 1 mm (**38**, **39**), 2 mm (**36**, **37**), 5 mm (**32–35**).

**Figures 40–44.**
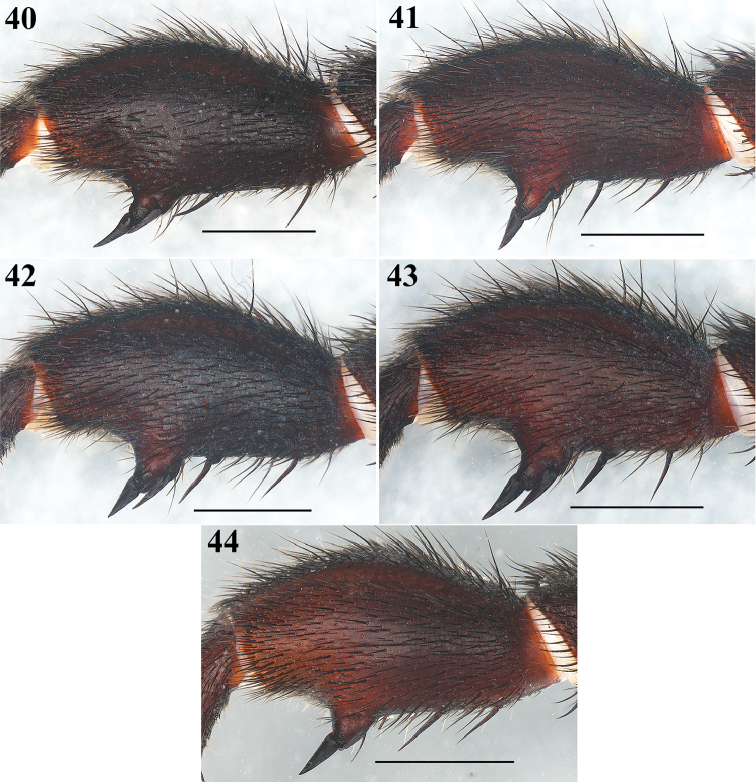
*Eucteniza zapatista* Bond & Godwin, 2013. Male: Morphological variation in the shape of the tibia I and macrospines of the five males collected for this study. Scale bars 2 mm.

**Figures 45–49.**
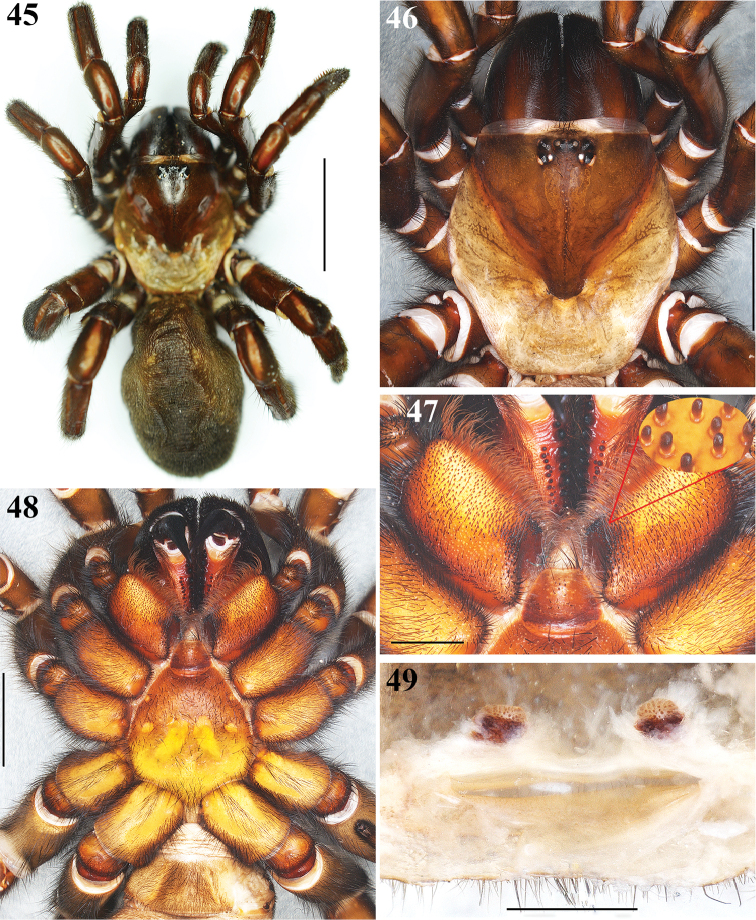
*Eucteniza zapatista* Bond & Godwin, 2013. Female: **45** Habitus dorsal **46** carapace, dorsal view **47** Endites, ventral view; detail of the cuspules **48** Prosoma, ventral view showing coxae, sternum, labium and endites **49** Spermathecae, dorsal view. Scale bars 1 mm (**49**), 2 mm (**47**), 5 mm (**46**, **48**), 10 mm (**45**).

**Figures 50–52.**
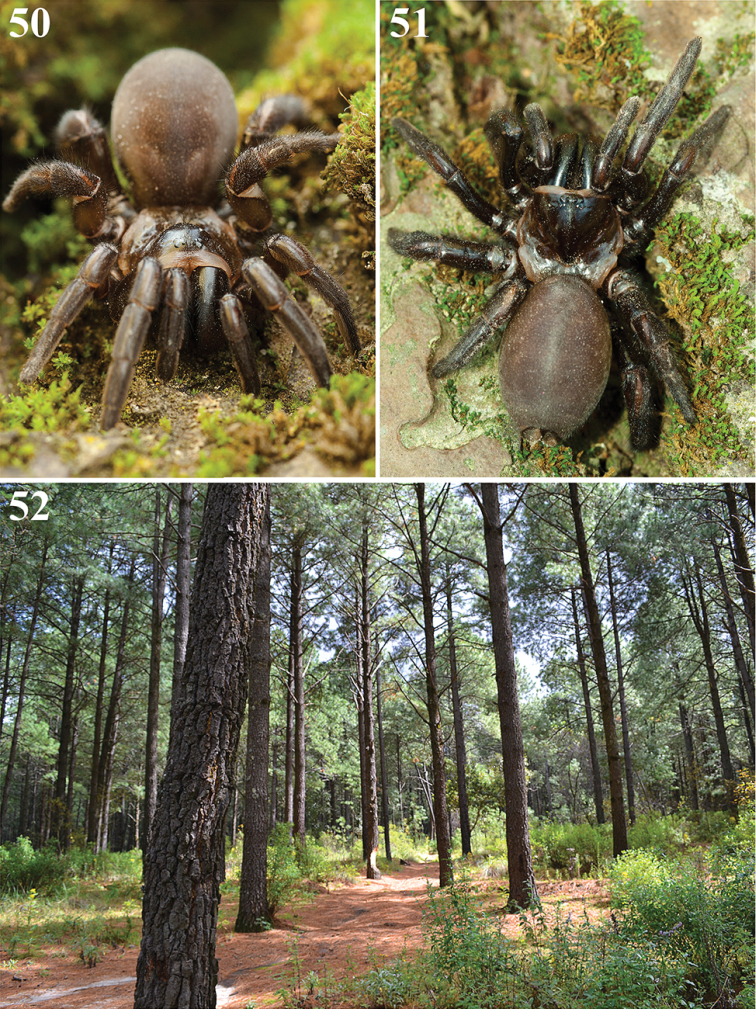
**50**, **51** Live female of *Eucteniza zapatista* Bond & Godwin, 2013 outside of her burrow (photos by Victor H. Jiménez Arcos) **52** Pine forest of the Parque Nacional la Malinche (PNLM), Tlaxcala, Mexico, where the specimens of *Eucteniza zapatista* used for this study were collected (photo by Alejandro Valdez Mondragón).

##### Redescription.

**Male**: Specimen collected using pitfall traps, preserved and observed in 80% ethanol. *Measurements*: Total length (prosoma + opisthosoma) 17.68. Carapace 8.30 long, 7.40 wide. Clypeus length 0.80. Diameter of AME 0.46, ALE 0.40, PME 0.25, PLE 0.30. Labium: LBl 0.88, LBw 1.31. Sternum: STRl 4.70, STRw 4.25. Leg lengths: I femur 7.70/ patella 4.30/ tibia 5.90/ metatarsus 5.10/ tarsus 3.10/ total 26.10; II- 7.00/ 3.40/ 5.20/ 5.50/ 3.20/ 24.30; III- 5.90/ 3.30/ 3.75/ 5.90/ 3.70/ 22.55; IV- 7.20/ 3.80/ 6.00/ 6.50/ 3.70/ 27.20. Leg formula: 4-1-2-3. *Prosoma*: Carapace longer than wide, protruding anteriorly, with surface smooth, setose in posterior part, hexagonal shaped, dark brown in anteriorly and lighter posteriorly (Figs 21, 23). Ocular region slightly elevated (Fig. 26). Foveal groove deep and procurved, U-shaped (Fig. 23). AER slightly procurved, PER recurved. Largest AME, smallest PME (Fig. 26). Sternum longer than wide, pyriform shaped, dark orange, setose, with sigilla (Fig. 24). Posterior sternal sigilla large and elongate, medial pair of anterior sigilla moderate in size, anterior pair small and marginal (Fig. 24). Labium wider than long, brown, with long setae anteriorly, without cuspules (Fig. 25). Endites long and setose, with an apical-prolateral inconspicuous conical apophysis, without cuspules (Fig. 25). *Chelicerae*: Promargin furrow with 7 teeth, retromargin furrow with approximately 18–20 small denticles. Rastellum consisting of 6-8 spines on a mound. *Opisthosoma*: Longer than wide, setose, gray, without pattern dorsally; lighter gray ventrally (Figs 21, 22). Spinnerets beige (Fig. 27). PMS small and rounded, single segment, with spigots. PLS long and conical, all 3 segments with spigots: basal segment length > median segment > distal segment. *Legs*: Very light tarsal scopulae on legs I, II, III (Fig. 39), absent in IV. Tibiae, metatarsi and tarsi with trichobothria, lacking pattern, only tarsi with slightly staggered dorsal row, variable in number. Legs spination pattern: Tibia I: with paired ventral megaspines (Figs 32, 33, 36) on prominent base (Fig. 38); tibiae II: with 1 thin and long megaspine (Figs 34, 35) on a slender base (Fig. 37); tibiae III and IV: scattered long spiniform setae; metatarsi and tarsi with scattered spination pattern. *Pedipalps*: Articles setose. Femora brown, long and cylindrical. Patellae brown, lighter ventrally (Figs 28, 29). Tibiae brown, lighter ventrally, long and cylindrical, widened in middle part (Figs 28, 29). Cymbium brownish and setose, without spines (Figs 28–30). Bulb oval, located toward internal part of the pedipalp (Figs 30, 31). Embolus short, thin and curved, pointing toward retrolateral part of the tibia (Figs 28–31).

##### Description.

**Female.** Similar to the male, differences: Specimen collected manually, preserved and observed in 80% ethanol. *Measurements*: Total length (prosoma + opisthosoma) 29.50. Carapace 12.20 long, 11.10 wide. Clypeus length 0.80. Diameter of AME 0.47, ALE 0.46, PME 0.23, PLE 0.43. Labium: LBl 1.31, LBw 1.87. Sternum: STRl 7.50, STRw 6.50. Leg lengths: I femur 9.10/ patella 5.10/ tibia 5.70/ metatarsus 4.30/ tarsus 2.50/ total 26.70; II- 7.60/ 5.10/ 4.80/ 4.00/ 2.20/ 23.70; III- 6.50/ 4.80/ 3.00/ 4.20/ 3.10/ 21.60; IV- 8.90/ 5.50/ 6.70/ 6.00/ 3.40/ 30.50. Leg formula: 4-1-2-3. *Prosoma*: Carapace markedly more anteriorly protruding than the male, small setae posteriorly, lighter brown anteriorly and posterior part markedly lighter color than the male (Figs 45, 46). Anterior part of ocular region more setose than the male. Sternum darker orange than the male (Fig. 48). Anterior pair of small and marginal sigilla more visible than the male (Fig. 48). Labium wider than long, brown, with long setae anteriorly, with nine cuspules (Fig. 47). Endites brown in retrolateral part, light orange toward prolateral part, with numerous cuspules (Fig. 47). *Chelicerae*: Promarginal furrow with nine teeth, retromarginal furrow with approximately 19 small denticles (Fig. 47). Rastellum consists of 5-7 spines on a mound. *Opisthosoma*: Setose, lighter gray toward anterior part, darker gray coloration than the male; ventrally, genital area dark brown (Fig. 48). Spinnerets dark brown. *Legs*: Short and stout legs compared with the male (Figs 45, 50, 51). Long and dense scopulae on metatarsi and tarsi I, II, absent in III and IV. Legs spination: Legs without megaspines; Tibiae I: v(1+1+1+1); tibiae II: v (1+1+1); tibiae III and IV: scattered long spiniform setae; metatarsi I and II: v(1+2); metatarsi III: v(2+2+1); metatarsi IV: scattered long spiniform setae; tarsi I and II: without spines; tarsi III and IV: with scattered spination pattern. *Pedipalps*: Articles stouter and darker coloration than the male, setose, with long and dense scopulae on tarsi. Tarsi with a single claw; spination pattern: v(1+1). Tibiae with scattered long spiniform setae. *Genital area*: Bulky, trapezoidal shape, setose, brown color (Fig. 48). Spermathecae with single oval bulbs, paired, with dark basal stalk, the bulbs seems to be porous (Fig. 49).

##### Variation.

Males (N = 5): Cl 6.8–8.3, 7.62±0.62; Cw 6.5–7.3, 6.84±0.38; STRl 4.30–4.90, 4.60±0.25; STRw 3.80–4.30, 4.00±0.19; PTl 3.50–4.20, 3.90±0.27; PTw 1.5–1.8, 1.62±0.13; Tibiae I (length): 4.60–5.90, 5.18±0.55. There is variation in the width of the tibia I and in the position of the ventral megaspines; in three specimens megaspines are close together and in two the megaspines are separated (Figs 40–44).

##### Natural history.

All specimens examined were collected in the PNLM, a temperate pine-oyamel forest at 3000-3250 m of elevation (Fig. 52). The four specimens (LATLAX-Ara0031) were collected using pitfall traps in a pine forest (Fig. 52). The specimen (LATLAX- Ara0033) was hand collected walking on the ground. The female (LATLAX- Ara0032) was hand collected from a vertical burrow of ~50-60 cm deep located at 2 m on a wall along road-cut in a pine forest, the female was also found in the bottom of the burrow (Figs 50, 51).

##### Distribution.

**Figure 53.**
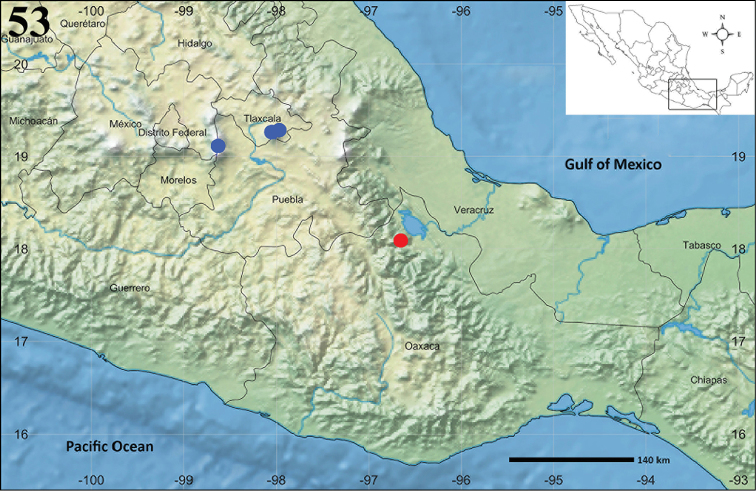
Known distribution records of *Aptostichus sabinae* sp. n. (red) (Oaxaca state), and *Eucteniza zapatista* Bond & Godwin, 2013 (blue) (Tlaxcala and Puebla states).

MEXICO: Puebla, Tlaxcala (Fig. 53).

## Discussion

In general, to collect trapdoor spiders is a difficult task, and few species have been described using both males and females. Most of them are described using few specimens or even only the male holotypes. The females are more difficult to collect due to their fossorial nature, whereas males, during certain seasons of the year wander, making pitfall traps the best method or technique so far for their collection. As Bond (2012) said: “the most of the species can be collected only during certain times of the year and collecting typically requires that the burrows be excavated, an activity that is often very time-consuming”. The best technique so far seems to be that one proposed by Bond (2012), where one must sometimes use a “scraping” technique to find burrows by removing the first few centimeters of topsoil, thereby exposing the silk lined burrow, however, this technique is not very effective in sandy desert habitats (Bond 2012: 6). In the case of the genus *Aptostichus*, the only way to find females in desert habitats seems to be after winter rains, when the spiders extend, or clean out their burrows, leaving a small mound of sand at the burrow entrance (Bond 2012).

Although the genus *Aptostichus* was expected to have a relatively restricted biogeographic distribution in the southwestern United States and Baja California peninsula in Mexico, where the species are found in different habitats ranging from Mediterranean climates to the arid Mojave and Colorado deserts (Bond 2012: figs 1–6); the genus seems to have a wide spread distribution in Mexico as well. The diversity of the genus in Mexico is unknown, and *Aptostichus sabinae* sp. n. represents the very first new species described from Mexico. Under sampled biogeographical provinces for this genus such as California and Baja California, Sonora, and the Mexican Montane biotic component and its provinces, as well as the Sierra Madre Occidental, Transmexican Volcanic Belt (TVB), Cuenca del Balsas, and the Sierra Madre del Sur where *Aptostichus sabinae* was collected, are some of the most biodiverse provinces in Mexico for different groups of mygalomorph (Mendoza 2014, Ortiz and Francke 2016) and araneomorph spiders (Valdez-Mondragón and Francke 2015).

As the genus *Aptostichus*, the genus *Eucteniza* in Mexico has been poorly collected in the Sierra Madre Occidental (Bond and Godwin 2013: fig. 1). Most species have been collected in Mexico, towards the Sierra Madre Oriental, three species in Baja California Sur, and a widespread species in Texas, United States. *Eucteniza* is currently composed of 14 species, most of which are described from the Sierra Madre Occidental. However, although three species have been recorded in the TVB including *Eucteniza zapatista*, this biotic province has been poorly collected, mainly towards temperate montane forests mountains of the states of Estado de México, Michoacán, North of Guerrero, Colima and Jalisco, where more collecting remains to be done. The TVB is located in the Mexican Transition Zone, a region of overlap between the Neartic and Neotropic biotic regions, which represents the most biodiverse region in North America (Halffter et al. 1995, Halffter 2003, Brooks 2005, Morrone 2005, 2014). The different vegetation types, altitude, and climates of the Mexican biotic components and their biogeographical provinces (Morrone 2004, 2005, 2014), hints at the possibility that the both genera have even greater diversity than currently described.

## Supplementary Material

XML Treatment for Aptostichus

XML Treatment for Aptostichus sabinae

XML Treatment for Eucteniza

XML Treatment for Eucteniza zapatista
